# Current Trends in NSAID Prescribing for Osteoarthritis and Axial Spondyloarthritis: Insights From Moroccan Rheumatologists Practices

**DOI:** 10.1002/hsr2.71764

**Published:** 2026-05-20

**Authors:** Imane El Binoune, Benyahia Zineb, Samira Rostom, Bouchra Amine, Rachid Bahiri

**Affiliations:** ^1^ Department of Rheumatology A, El Ayachi Hospital Ibn Sina University Hospital, Mohammed V University Rabat‐Salé Morocco

## Abstract

**Background and Aims:**

Nonsteroidal anti‐inflammatory drugs (NSAIDs) remain the cornerstone of symptomatic management in osteoarthritis (OA) and axial spondyloarthritis (axial SpA), but safety concerns have reshaped prescribing behaviors. This study aimed to describe current NSAID prescribing practices among Moroccan rheumatologists for both conditions.

**Methods:**

A cross‐sectional descriptive survey was conducted among rheumatologists from public and private sectors. Data were collected using a 30‐item validated questionnaire approved by an expert committee.

**Results:**

Eighty‐two rheumatologists participated. More than half (56.1%) reported having no absolute age limit for NSAID use, while 24% avoided NSAIDs in patients aged > 75 years. The most concerning combination was NSAIDs with vitamin K antagonists (96.3%), and the most cited contraindication was active gastroduodenal ulcer (85.4%). Only 13.4% routinely requested renal function tests before prescribing NSAIDs. Proton pump inhibitors were co‐prescribed mainly for a history of gastrointestinal bleeding (93.9% with conventional NSAIDs; 87.8% with coxibs). Naproxen (95.1%), celecoxib (63.4%), and meloxicam (58.5%) were considered the safest for cardiovascular risk. Celecoxib was the first‐line NSAID for OA (47.6%), while diclofenac was preferred for axial SpA (34.9%) and celecoxib in patients with inflammatory bowel disease (73.2%). Nearly half (47.6%) prescribed NSAIDs on an as‐needed basis.

**Conclusion:**

NSAID prescribing in OA and axial SpA is increasingly personalized, with choices guided by comorbidities and safety concerns. Celecoxib is preferred for OA, while diclofenac remains first‐line for axial SpA, with caution in patients at gastrointestinal or cardiovascular risk.

## Summary

- This study provides an overview of current NSAID prescribing practices among Moroccan rheumatologists for osteoarthritis and axial spondyloarthritis.
- Celecoxib is the most commonly prescribed NSAID for osteoarthritis, while diclofenac remains the first‐line choice for axial spondyloarthritis.
- Prescription decisions are primarily guided by clinical symptoms such as pain and flare‐ups, rather than radiographic stage.
- There is a cautious approach to NSAID use in elderly patients and those with comorbidities, especially regarding gastrointestinal and renal risks.
- The findings emphasize the need for individualized NSAID therapy, balancing efficacy with safety concerns.

## 1 Introduction

Musculoskeletal disorders (MSDs) represent a significant burden to healthcare systems worldwide due to their high prevalence and significant impact on patients' quality of life. Osteoarthritis and axial spondyloarthritis (axSpA) stand out as common rheumatological conditions. Osteoarthritis, a degenerative joint disease, primarily affects elderly patients and is often responsible for chronic pain and functional limitations [1]. In contrast, axSpA, a chronic inflammatory disease of the spine and sacroiliac joints, typically affects younger patients and can lead to progressive ankylosis if not adequately managed [2].

Nonsteroidal anti‐inflammatory drugs (NSAIDs) play a crucial role in the symptomatic management of both conditions by reducing pain and inflammation. However, despite their efficacy, they are associated with significant adverse effects, particularly in the gastrointestinal, cardiovascular, and renal systems. These side effects limit their long‐term use and prompt healthcare professionals to adopt more individualized treatment strategies, taking into account the specific risks of each patient [3].

In addition, recent clinical guidelines and new safety data on NSAIDs have led to evolving prescribing practices that influence therapeutic decisions in the management of osteoarthritis and axial SpA [4]. These recommendations emphasize the importance of a thorough risk‐benefit assessment and the need to optimize treatment while minimizing adverse outcomes [5].

In this context, our study aims to explore and analyze current NSAID prescribing practices for osteoarthritis and axSpA to better understand the factors influencing these therapeutic decisions and identify potential disparities in the management of these conditions in Morocco.

## 2 Materials and Methods

### 2.1 Study Design and Population

We conducted a descriptive cross‐sectional survey of Moroccan rheumatologists working in both the public and private sectors. The study was conducted online using a structured questionnaire sent by email to a sample of 299 practitioners through professional channels. A brief introduction explaining the objectives of the study was included in the invitation. Participation was voluntary and responses were anonymous.

### 2.2 Data Collection

Data were collected using Google Forms, which allowed quick and easy access to the questionnaire. The form consisted of 30 single‐ and multiple‐choice questions divided into three main sections:

Practitioner characteristics: age, seniority, practice sector (public/private).

General NSAID prescribing habits: reasons for prescribing, preferred types of NSAIDs, criteria for choice.

Specific NSAID prescribing: one section dedicated to the use of NSAIDs in osteoarthritis and another to their use in axSpA.

### 2.3 Ethical Approval and Consent

As this study involved an anonymous questionnaire administered to healthcare professionals (rheumatologists) and did not include patient data, the Institutional Review Board (IRB) of [Name of Institution] determined that formal ethical approval was not required. Participation was voluntary, and informed consent was implied by completion of the questionnaire.

### 2.4 Statistical Analysis

Data were analyzed using SPSS software, version 13.0. Normally distributed quantitative variables were described by their means and standard deviation, while variables with an abnormal distribution were expressed as median with interquartile range (IQR, 25th–75th percentiles). Categorical variables were presented as absolute (n) and relative (%) frequencies. The study is descriptive and based on categorical data. We reviewed the Assel et al. guidelines and ensured that statistical reporting was consistent with these recommendations.

## 3 Results

A total of 98 out of 299 invited rheumatologists completed the survey, yielding a response rate of 32.8%. Among the respondents, 78% were women, with a mean age of 47  ± 17 years and a mean professional experience of 10 years. Half of the participants (50%) worked in the private sector, while the remainder practiced in the public sector.

**Figure 1:**
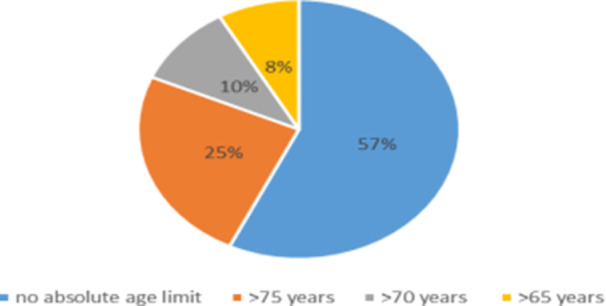
Age at which NSAIDs are no longer prescribed to patients.

**Figure 2:**
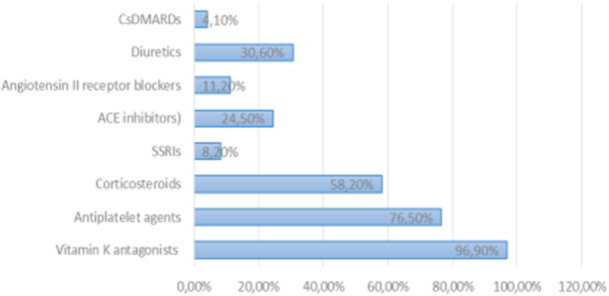
Most cautious drug combinations in NSAID prescriptions.

Regarding NSAID prescribing practices, the majority of rheumatologists (57.1%) did not have an absolute age limit for NSAID use, whereas 24.5% avoided prescribing them to patients older than 75 years (Figure 1). The combination of NSAIDs with vitamin K antagonists (VKAs) was considered the most high‐risk drug interaction, reported by 96.9% of participants (Figure 2). Among contraindications, active peptic ulcer was cited most frequently (85.4%).

In terms of monitoring, only 13.4% of rheumatologists systematically requested renal tests before prescribing NSAIDs. For gastroprotection, the main reason to combine NSAIDs with a proton pump inhibitor (PPI) was a history of gastrointestinal bleeding, cited by 93.9% of respondents for conventional NSAIDs and 87.8% for coxibs.

**Figure 3:**
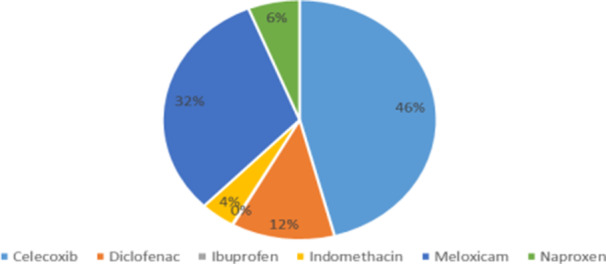
First‐line NSAID prescribed for osteoarthritis.

Regarding cardiovascular safety, naproxen (95.1%), celecoxib (63.4%), and meloxicam (58.5%) were considered the best‐tolerated NSAIDs. In osteoarthritis, celecoxib was the first‐choice NSAID for 45.9% of respondents (Figure 3). While 73.2% did not consider radiographic stage when prescribing, the presence of a congestive flare (96.3%) and pain intensity according to VAS (78%) were key decision‐making factors. Intra‐articular NSAID injections were rarely used, reported by only 2.4% of participants.

**Figure 4:**
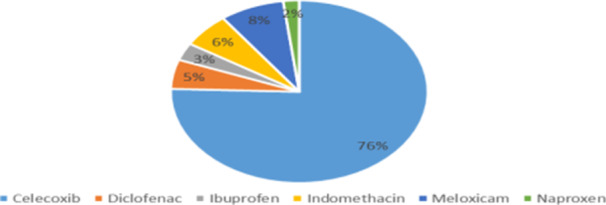
First‐line NSAID prescribed for IBD associated with axial SpA.

For axSpA, diclofenac (34.9%) was the most commonly prescribed first‐line NSAID, whereas celecoxib was preferred in patients with associated inflammatory bowel disease (IBD) (75.5%; Figure 4). Almost half of rheumatologists (47.6%) prescribed NSAIDs on demand, and 70.7% started treatment at the maximum recommended dose. Only 13.4% reported prescribing supramaximal doses.

Concerning treatment evaluation, 59.2% of rheumatologists tried three different NSAIDs before deeming a patient refractory, with 40.2% considering a maximum treatment duration of 20 days. In cases of associated IBD, 56.1% consulted a gastroenterologist before prescribing, while 12.2% systematically avoided NSAID use. Finally, 65.9% routinely screened for toxic nephropathy as part of adverse effect monitoring.

## 4 Discussion

### 4.1 General NSAID Prescribing Habits

The finding that 56.1% of physicians do not set an absolute age limit before prescribing NSAIDs is notable. Elderly patients (≥ 65 years) are generally considered at higher risk of adverse effects, including gastrointestinal, renal, and cardiovascular complications. Recent reviews emphasize increased caution in older patients, recommending dose adjustments, careful monitoring, and individualized assessment, without imposing a strict age cutoff [5, 6]. The French Society of Rheumatology (SFR) also recommends careful monitoring, especially regarding cardiovascular risk, and prescribing NSAIDs at the lowest effective dose for the shortest possible duration [7].

Regarding cardiovascular tolerability, our results align with the literature. Naproxen is frequently regarded as having a favorable cardiovascular profile [8]. Although celecoxib has been associated with an increased cardiovascular risk in older studies, more recent trials indicate that at moderate doses, celecoxib is not inferior to ibuprofen or naproxen regarding cardiovascular safety [8, 9]. Etoricoxib, another selective COX‐2 inhibitor, has also shown efficacy in pain management. Meta‐analyses comparing coxibs (celecoxib, rofecoxib, etoricoxib, lumiracoxib) and high‐dose traditional NSAIDs (diclofenac 150 mg, ibuprofen 2400 mg, naproxen 1000 mg) suggest an increased risk of major vascular events with some coxibs, though the magnitude varies across drugs [10]. Meloxicam, a partially selective COX‐2 inhibitor, generally carries a lower cardiovascular risk than non‐selective NSAIDs such as ibuprofen [10].

Gastrointestinal toxicity remains a major concern. Pooled analyses of 9461 patients aged ≥ 65 years in randomized trials comparing celecoxib (200–400 mg/day) to non‐selective NSAIDs (naproxen, ibuprofen, diclofenac) showed that celecoxib was better tolerated, with fewer gastrointestinal complications [11]. Naproxen is also considered better tolerated than other non‐selective NSAIDs due to its longer half‐life, which reduces chronic high‐dose exposure; however, the risk of gastrointestinal bleeding persists with long‐term use [12].

### 4.2 Osteoarthritis (OA)

#### 4.2.1 Choice of NSAID

The OARSI guidelines recommend oral NSAIDs for patients with knee, hip, or polyarticular OA without comorbidities, preferring non‐selective NSAIDs combined with a PPI or selective COX‐2 inhibitors [4, 13]. NSAIDs are contraindicated in patients with cardiovascular comorbidities unless strictly necessary, in which case drugs with a favorable safety profile should be prescribed at the lowest effective dose for the shortest duration [13]. Our results reflect these recommendations, showing widespread use of celecoxib and careful selection based on patient risk.

#### 4.2.2 Prescription Duration

More than half of the rheumatologists in our survey prescribed NSAIDs for no more than 7 days, consistent with SFR, EULAR, and OARSI recommendations [7, 13, 14]. Duration should be adjusted according to clinical evolution, and alternative therapies considered to minimize prolonged NSAID use.

#### 4.2.3 Radiographic Stage and NSAID Prescription

Most physicians did not consider radiographic stage when prescribing NSAIDs, relying instead on pain intensity (VAS) and the presence of symptomatic flares. This approach aligns with current evidence, which shows inconsistent correlation between radiographic severity and pain [15, 16]. These findings highlight the need for individualized treatment based on clinical assessment rather than imaging alone.

#### 4.2.4 Intra‐Articular NSAID Use

Only 2.4% of rheumatologists reported using NSAIDs intra‐articularly. Most guidelines do not support this practice due to limited evidence of efficacy. RCTs comparing intra‐articular ketorolac with corticosteroids in knee OA found similar short‐term pain relief, though sample sizes were small and long‐term cartilage effects remain unclear [17, 18, 19]. In resource‐limited settings or in patients with corticosteroid contraindications, intra‐articular NSAIDs may offer a pragmatic alternative, but further research is required to clarify safety and efficacy.

### 4.3 Axial Spondyloarthritis (axSpA)

Our findings are broadly in line with ASAS‐EULAR recommendations (2022) advocating NSAID use only when needed to control symptoms, with preference for on‐demand therapy to reduce long‐term gastrointestinal and cardiovascular risks [5, 20]. Indomethacin remains commonly used due to its anti‐inflammatory potency, although recent studies show similar efficacy and better tolerability with other NSAIDs, such as diclofenac and celecoxib [21]. Our study confirms that diclofenac is widely used in practice, with 79.6% of rheumatologists including it among their top three first‐line choices. These findings suggest that availability, cost‐effectiveness, and clinical experience continue to influence prescribing behavior [21, 22].

#### 4.3.1 NSAID Use in AxSpA With IBD

Managing patients with axSpA and chronic IBD is challenging due to potential exacerbation of bowel inflammation. Literature suggests NSAIDs may trigger flares in Crohn's disease but less so in ulcerative colitis [5, 23, 24, 25]. Our survey shows that 76% of physicians preferentially prescribe celecoxib, reflecting a strategy of using selective NSAIDs and sparing therapy in high‐risk patients. This practice aligns with current EULAR and ACR recommendations emphasizing individualized, cautious use.

### 4.4 Study Strengths

This study provides a representative view of NSAID prescribing practices in Morocco across public and private sectors. The questionnaire was validated by a panel of five senior rheumatologists, ensuring content validity. The study allows comparison of prescribing practices in degenerative (OA) and inflammatory (axSpA) conditions and assesses adherence to guideline recommendations. It also highlights challenges in adjusting NSAID doses for comorbidities and monitoring for adverse effects.

### 4.5 Study Limitations

The study has several limitations. The sample size (98 rheumatologists) is relatively small, limiting generalizability. The low response rate (98/299) may introduce non‐response bias. The online survey format could favor respondents with higher engagement or digital literacy, introducing selection bias. Additionally, this study is primarily descriptive and does not perform inferential analyses to explore associations between physician demographics and prescribing patterns. Future studies could expand to larger, international populations and incorporate inferential statistical analyses.

## 5 Conclusion

Our study provides insight into current NSAID prescribing practices for osteoarthritis and axial spondyloarthritis (SpA) among Moroccan rheumatologists. Celecoxib is frequently preferred as a first‐line NSAID for osteoarthritis, reflecting its relatively favorable cardiovascular and gastrointestinal safety profile, whereas diclofenac remains the most commonly used first‐line NSAID for SpA. Despite a similar efficacy of NSAIDs for mechanical and inflammatory conditions, careful consideration of patient comorbidities, risk factors, and potential adverse effects is essential to ensure optimal individualized therapy. Additionally, our findings highlight areas for improvement, such as systematic monitoring and adherence to guideline recommendations. Future studies could explore interventions to optimize NSAID prescribing, evaluate long‐term outcomes, and compare practices across different regions or healthcare systems.

## Author Contributions

**Imane El Binoune:** conceptualization, supervision, validation. **Benyahia Zineb:** data curation, formal analysis, investigation, methodology, writing – original draft, writing – review and editing. **Samira Rostom:** supervision, validation. **Bouchra Amine:** supervision, validation. **Rachid Bahiri:** supervision, validation.

## Disclosure

The lead author Benyahia Zineb affirms that this manuscript is an honest, accurate, and transparent account of the study being reported; that no important aspects of the study have been omitted; and that any discrepancies from the study as planned (and, if relevant, registered) have been explained.

## Conflicts of Interest

The authors declare no conflicts of interest.

## Data Availability

The authors confirm that the data supporting the findings of this study are available within the article and its supporting materials.
